# Persistence of Neuronal Alterations in Alcohol-Dependent Patients at Conclusion of the Gold Standard Withdrawal Treatment: Evidence From ERPs

**DOI:** 10.3389/fpsyt.2021.666063

**Published:** 2021-08-30

**Authors:** André Kuntz, Pascal Missonnier, Anne Prévot, Grégoire Favre, François R. Herrmann, Damien Debatisse, Marco C. G. Merlo, Isabelle Gothuey

**Affiliations:** ^1^Mental Health Network Fribourg (RFSM), Sector of Psychiatry and Psychotherapy for Adults, Marsens, Switzerland; ^2^Laboratory for Psychiatric Neuroscience and Psychotherapy, Department of Neuroscience, Faculty of Science and Medicine, University of Fribourg, Fribourg, Switzerland; ^3^School of Health Sciences (HEdS-FR), HES-SO University of Applied Sciences and Arts Western Switzerland, Fribourg, Switzerland; ^4^Division of Geriatrics, Department of Rehabilitation and Geriatrics, Geneva University Hospitals and University of Geneva, Thônex, Switzerland; ^5^HELIOS Privatkliniken GmbH – Wuppertal-Universität/Barmen, Wuppertal, Germany; ^6^Department of Neurosurgery, Universität Kliniken der Stadt Köln gGmbH, Krankenhaus Merheim, Cologne, Germany

**Keywords:** alcohol addiction, event-related potentials, benzodiazepine withdrawal treatment, P3b component, N1 component, N2 component

## Abstract

**Background:** One of the main challenges for clinicians is to ensure that alcohol withdrawal treatment is the most effective possible after discharge. To address this issue, we designed a pilot study to investigate the efficacy of the rehabilitation treatment on the main stages of information processing, using an electroencephalographic method. This topic is of main importance as relapse rates after alcohol withdrawal treatment remain very high, indicating that established treatment methods are not fully effective in all patients in the long run.

**Method:** We examined in alcohol-dependent patients (ADP) the effects of the benzodiazepine-based standard detoxification program on event-related potential components at incoming (D0) and completion (D15) of the treatment, using tasks of increasing difficulty (with and without workload) during an auditory oddball target paradigm. Untreated non-alcohol-dependent-volunteers were used as matching controls.

**Results:** At D0, ADP displayed significantly lower amplitude for all ERP components in both tasks, as compared to controls. At D15, this difference disappeared for the amplitude of the N1 component during the workload-free task, as well as the amplitude of the P3b for both tasks. Meanwhile, the amplitude of the N2 remained lower in both tasks for ADP. At D0, latencies of N2 and P3b in both task conditions were longer in ADP, as compared to controls, whilst the latency of N1 was unchanged. At D15, the N2 latency remained longer for the workload condition only, whereas the P3b latency remained longer for the workload-free task only.

**Conclusion:** The present pilot results provide evidence for a persistence of impaired parameters of ERP components, especially the N2 component. This suggests that neural networks related to attention processing remain dysfunctional. Longitudinal long-term follow-up of these patients is mandatory for further assessment of a link between ERP alterations and a later risk of relapse.

## Introduction

Alcohol addiction has been associated with a wide range of temporary cerebral and cognitive deficits related to acute or permanent alcohol intoxication. These neuronal dysfunctions have gained considerable importance as critical features of mental illness (1, 2). In a recent study, cognitive control deficits were discussed as dependent biomarkers (3). Therefore, it is now believed that neurocognitive deficits may provide valid indexes to measure the clinical evolution of the disease (4). Moreover, a systematic assessment of the functioning of the neural bases supporting the main cognitive functions appears essential whilst monitoring detoxification, as well as on completion of the treatment. In this context, complete recovery of cognitive functions following an alcohol withdrawal treatment could be a positive prognostic factor for long-term abstinence.

The management of treatment of alcohol-dependent patients (ADP) with alcohol withdrawal syndrome is heterogeneous, with various drugs or drug combinations being used as pharmacotherapies (5). Benzodiazepines (BZ) are considered to be a gold standard in alcohol withdrawal with an ability to reduce the risk of epileptic seizures and delirium tremens as potential complication during an alcohol withdrawal (6, 7). From a pharmacological point of view, chronic alcohol consumption modifies the structure of γ-aminobutyric acid type A (GABA_A_) receptors (8). This leads to a decrease in inhibitory GABAergic neurotransmission, resulting in an imbalance between excitation and inhibition (9). Benzodiazepines bind to GABA_A_ receptors and increase the number of these receptors, which restores the neuronal efficiency, as well as the functionality of cortical neural networks that underlie cognitive processes (10, 11). The BZ selected as withdrawal treatment in our study was Oxazepam®, which is a short-to-intermediate-acting BZ, with an elimination half-life of 3–21 h and a metabolization via glucuronidation outside the liver. Therefore, administration of this drug may be safer with patients suffering from hepatic problems, such as ADP (12).

Cognitive functioning in ADP is usually investigated using questionnaires, behavioral tasks, or a combination of these methods. They allow to clarify the type and intensity of the cognitive deficit, and to establish a pattern of alcohol-related cognitive damage. However, they provide no information on the mechanisms of cerebral deficiencies during specific cognitive tasks. In contrast, functional magnetic resonance imaging (fMRI) methods have an excellent spatial resolution, which offers the opportunity to visualize brain activity and to identify the neural circuits involved in a cognitive task. However, although fMRI is particularly suitable for studying the impact of drugs on the dynamics of neural networks, its temporal resolution is not sufficient to explore brain activation patterns during sensory and cognitive processes occurring in short time ranges (hundreds of milliseconds). This is the main reason why we chose event-related potentials (ERPs), as they can explore the functional activation of neocortical circuits with a high temporal resolution, and thus represent a more sensitive method in this context. ERPs represent a series of EEG events that reflect the progressive activation of neural subpopulations in the course of cognitive processing. In the 0–150 ms after stimulus onset, ERPs are related to the physical characteristics of the stimuli (i.e., exogenous ERPs) and reflect the sensory treatment, whereas the later ERPs are task-dependent (i.e., endogenous ERPs). Typically, the auditory oddball task that consists of detecting infrequent (i.e., target) amongst frequent stimuli (i.e., standards) allows observing both exogenous and endogenous ERPs. Interestingly, significant changes on main endogenous ERPs were observed whilst combining an oddball procedure with tasks of incrementally-varying difficulty, revealing that this protocol is well-adapted to investigate sensory, attention and memory/decision processes (13).

Previous EEG contributions have reported that ERP components spanning various stages of the information-processing stream can be altered by alcohol consumption (14). The component most widely used in alcoholism research is the P3b component (14), which is located in the centro-parietal region within 300–500 ms after stimulus onset, and is elicited in a wide range of paradigms involving detection of behaviorally-relevant targets (15, 16). It reflects processes related to working memory and contextual updating (17, 18). Latency increase and amplitude reduction of the P3b are consistently reported in patients with chronic alcohol abuse (18, 19).

The P3b component is preceded by the perceptive-auditory N1 and the endogenous N2 components (20, 21). The N2 is evoked by an odd physically deviant auditory stimulus that occurs in a series of frequent standard stimuli, therefore it is related to the activation of neural networks involved in the allocation of attention to novel stimulus. For its part, the N1 is associated with attention-catching properties and represents the phase of sensory gating (22). A reduction of the amplitude of these two components has been described in active alcoholic use (23).

The aim of the present study was to detect differences in ERPs between ADP and controls at inclusion, as well as a significant improvement after BZ treatment. Therefore, we carried out an EEG activation pilot study associated with endogenous and exogenous ERPs analysis during tasks requiring upgrading of cognitive resources in ADP at incoming (first recording, D0) and outgoing (second recording, D15) of the standard pharmacological alcohol withdrawal treatment program, as compared to healthy non-alcohol-dependent volunteers (controls). We focused on the analysis of specific ERP components (N1, N2, and P3b) known to be relevant in the context of the alcohol withdrawal treatment.

## Materials and Methods

### Participants

Eighteen alcohol-dependent inpatients who wanted to stop abusing alcohol were included in the study (4 women, 14 men; mean age: 44.9 (± 10.5 SD) years; age range: 29–59) (see Table 1). Inpatients were diagnosed with alcohol dependence according to Diagnostic and Statistical Manual of Mental Disorders (DSM)-IV criteria (24). They were recruited in the specialized program for adult detoxification of the Mental Health Network Fribourg (RFSM), Fribourg/Freiburg, Switzerland.

**Table 1:** Demographic and clinical characteristics at D0 of controls (*n* = 18) and alcohol-dependent patients (ADP) (*n* = 18).

**Demographic**	**Clinical**				
		**Controls**	**ADP**	***t***	***p*-value**
		**Mean (SD**	**Mean (SD)**		
Age (years)		44.8 (9.3)	44.9 (10.5)	0.64	0.32
Sex (male: female ratio)		12:6	14:4	0.05	0.96
Laterality (right: left ratio)		18:0	18:0		–
	AAAQ (approach)		33.4 (11.5)		
	AAAQ (avoidance)		49.4 (11.5)		
	Alcohol use		8.5 (3.9)		
	AUDIT		27.6 (6.6)		
	BDI		18.6 (11.9)		
	BIS (attentional)		18.3 (4.9)		
	BIS (motor)		23.9 (4.0)		
	BIS (non-planning)		26.7 (4.3)		
	STAI (state)		46.2 (17.1)		
	STAI (trait)		49.1 (13.4)		

*AAAQ, approaches and avoidance of alcohol questionnaire; Alcohol use, the alcohol consumption timeline follow-back method; AUDIT; the alcohol use disorders identification Test; BDI, beck depression inventory; BIS, barratt Impulsivity Scale; STAI, the state-trait anxiety inventory*.

*Scores without unit*.

*Data are presented as mean (SD)*.

At inclusion, all subjects were diagnosed based on a clinical evaluation made independently by two trained psychiatrists. Additionally, we performed the following standardized questionnaires covering alcohol consumption, depression, anxiety, and impulsiveness: the Beverage Rating Scale (25), the Drinking History Questionnaire (26), the Alcohol consumption assessment self-measure (27), the Alcohol use disorder identification test AUDIT (28), the Approach and Avoidance of Alcohol Questionnaire [AAAQ, (29)], and Beck Depression Inventory scale [(BDI-II, (30)]. Subjects were also evaluated with a clinical and socio-demographical examination, the Barrat Impulsiveness Scale-11 (BIS-11), and State-Trait Anxiety Inventory for adults form Y [STAI form Y, (31)]. Inpatient treatment included 2–3 consultations per week (medical and nurse staff) with elements of psychoeducation and psychotherapy, 1 session of specialized group therapy (either music therapy, ergotherapy, art therapy or movement therapy). As expected in the alcohol-dependent population, 16 out of 18 patients had psychiatric comorbidities; 5 out of 18 patients suffered from a comorbid addictive disorder. Consequently, patients received psychotropic medications for their comorbid psychiatric disorders (see Table 2).

**Table 2:** Psychiatric clinical use characteristics of alcohol dependent patients (ADP).

	**ADP (*n*)**	**Mean (SD)**	**Range**
**Psychiatric and addiction comorbidities**
Mild to moderate depressive episode (ICD 10 F32 & 33)	9		
Bipolar depression (ICD 10 F 31)	2		
Personality disorder (ICD 10 60.3 & 61)	3	
Cannabis abuse (ICD 10 F 12.1)	1		
Cannabis addiction (ICD 10 F 12.2)	2		
Cocaine addiction (ICD 10 F 14.2)	1		
Combined psychiatric and addiction comorbidity	2		
**Doses of Oxazepam in mg/day**
D0 (mg/day)		89.17 (67.2)	60–240
D15 (mg/day)		16.87 (19.2)	0–45
Dose reduction in % D15-D0		80.52 (18.8)	50–100
**Duration of alcohol consumption before treatment**
Duration of addictive alcohol consumption			
<3 years	2		
> 3 years	16		
**Drinking pattern before treatment**
Daily	17		
Episodic drinking on weekends	1		
**Quantity of alcohol consumption before treatment**
Daily alcohol consumption in SDU		20.6 (13.6)	10–55

*Standard drinking unit (SDU). 1 standard drinking unit corresponds to 10-12 g of pure alcohol (in Switzerland)*.

We also included 18 healthy non-alcoholic volunteers acting as controls matched in age and education level (6 women, 12 men; mean age: 44.8 (± 9.3 SD) years; age range: 33–61) (see Table 1), without any history of sustained head injury or other neurological or psychiatric disorders. Psychiatric disorders were excluded using the Mini-International Neuropsychiatric Interview (MINI) (32) conducted by a trained psychiatrist. Moreover, none exhibited alcohol or drug abuse. Those with regular use of psychotropic drugs, stimulants or β-blockers were not included.

The protocol was conducted using a within-subject design so that all participants were recorded twice: (1) Day 0 = D0: ingoing for ADP, and first recording for controls; (2) Day 15 = D15: outgoing of the withdrawal treatment 14.1 ± 2.0 days after ingoing for ADP, and second recording after 14.5 days ± 1.9 for controls (mean ± SD).

The initial mean standard dose of benzodiazepines (Oxazepam®) was 89.17 ± 67.18 mg/day (range 60–240 mg/d, depending on the degree of withdrawal symptoms) with a progressive reduction from the 4th day onwards. Twelve ADP still received Oxazepam® at D15 with a mean dose of 18.41 ± 19.37 mg/day, corresponding to a reduction of 72.47 ± 36.33% (see Table 2 for additional information).

All participants were tested at D15 with the extensive neuropsychological CogState Battery (see Table 3 for details). All participants had normal or corrected-to-normal visual acuity and none of them suffered from a severe physical impairment. All were right-handed. The study was approved by the Ethics Committee of the University of Fribourg, Switzerland, and the study protocol was in line with the Helsinki Declaration. All subjects provided written informed consent at inclusion.

**Table 3:** CogState neuropsychological performances for controls (*n* = 18) and alcohol-dependent patients (ADP) (*n* = 18) at D15.

**Tasks**	**Controls Mean (SD)**	**ADP Mean (SD)**	***P*-value**
**Executive function**
Set-shifting task—ER	15.6 (9.3)	22.0 (10.7)	0.09
**Executive function/Spatial problem solving**
Groton maze learning test—ER tot	40.4 (12.7)	47.1 (22.6)	0.35
**Psychomotor function/Speed of processing**
Detection task speed, log_10_(ms)	2.5 (0.1)	2.5 (0.1)	0.49
**Visual attention/Vigilance**
Identification task speed, log_10_(ms)	2.7 (0.0)	2.8 (0.1)	0.05
**Visual learning and memory**
Groton maze learning test—DR	6.7 (5.9)	7.6 (6.6)	0.70
**Verbal learning and memory**
International shopping list			
- CRtot	29.0 (3.3)	24.6 (4.8)	0.01
- DR	10.3 (1.7)	8.6 (2.7)	0.06
**Working memory**
One back task—AP	1.3 (0.2)	1.2 (0.2)	0.49
**Social cognition**
Social-emotional cognition task—AP	1.2 (0.1)	1.2 (0.2)	0.98

*Data are presented as mean (SD). AP, accuracy of performance (arcsine transformation of the square root of the proportion of correct responses); ER tot, total number of errors; DR, delayed recall (number of correct responses); CR tot, total number of correct responses. See text for details*.

### Experimental Design

The subjects were seated in a comfortable chair in a sound- and light-attenuated room while listening to the stimuli presented through loud speakers. Stimuli were binaurally presented to each ear. They consisted of pure sine-wave tones including standard, or “frequent” (80%), low-pitch tones (1000 Hz), and deviant targets, or “infrequent targets” (20%), high pitch tones (2000 Hz). Frequent and infrequent target tones were presented in a random order. Infrequent tones were all isolated. Sound intensity was adjusted for each subject at the beginning of the experiment, in order to obtain the same subjective loudness at both ears. In practice, these values ranged between 80 and 90 dB SPL. Each tone lasted 100 ms with a 10 ms rise and fall time. Inter-stimulus intervals randomly varied between 800 and 1200 ms. Subjects were instructed to remain quiet and to only move their right index finger in accordance with the nature of the task in order to minimize muscle artifacts.

Two different tasks were tested in the following order: (1) for the simple detection task used as the control task, participants were instructed to respond as quickly as possible by pressing a button with their right index finger as soon as they detected an infrequent target; (2) for the counting backward task, participants not only had to press the button, but were also asked to subtract 1 day to the precedent starting from the date of the experiment (i.e., counting in reverse) for each infrequent target detected. For non-target frequent trials no response was required, neither motor, nor counting. Thus, mental workload increased from a workload-free task to a counting backward (i.e., highly demanding workload) task.

Each task was tested in a unique session composed of 200 sequential stimuli. Subjects were informed about the nature of the forthcoming task before each sequence. For both conditions, subjects were asked to keep their eyes closed and to avoid blinking and eye movements. The total duration of the experiment was ~30 min. Reaction times (RTs) and performance were systematically recorded, but no feedback on performance was provided. Electrophysiological and neuropsychological assessments were performed in the morning.

### Electrophysiological Recordings

In order to identify easily applicable EEG markers in routine clinical settings, continuous EEG (Advanced Neuro Technology Company-ANT, The Netherlands) was recorded using 21 surface electrodes placed over the scalp according to the 10–20 international electrode placement system (33). Linked mastoid right and left electrodes were used as a reference to respect equidistance between electrodes in order to achieve a central equipotency of the upper half sphere for later source reconstruction of ERPs. Skin impedance was kept below 5 kΩ. Physiological signals were sampled at 256 Hz, the lower cut-off was 0.03 Hz and the upper cut-off was 100 Hz (DC amplifiers, ANT). Right, left, supra-, and infraorbital electrodes monitored horizontal and vertical electro-oculograms. Simultaneously to stimuli onsets (frequent or infrequent targets), TTL-pulses (Transistor-Transistor Logic) were sent to the EEG-recording system. These TTL-pulses were used off-line to segment the continuous EEG-data into epochs, synchronized with stimulus onset.

### Data Processing

Data were analyzed with the Advanced Source Analysis software (Advanced Source Analysis [ASA] 4.0 software, ANT, The Netherlands). After artifact removal and off-line correction of ocular artifacts (voltage step > 70 μV/ms or peak to peak deflection within 200-ms intervals > 200 μV/ms), data from trials with correct answers were averaged according to task conditions (control or backward counting) and stimuli tones (frequent or infrequent target). EEG data were averaged over a window of 700 ms with a 200 ms pre-stimulus onset and band-pass filtered between 0.1 Hz (24 dB/octave) and 30 Hz. Responses were finally averaged with a 200-ms baseline epoch prior to stimulus onset.

#### ERPs

ERPs corresponding to correct answers in infrequent target tones were thereafter analyzed for each condition and group. The ERP components of interest were the exogenous N1 component and the endogenous N2, and P3b components. These components were identified in the grand-average waveform.

The component analyses were restricted to a cluster of central electrode locations (C3, Cz, and C4 electrodes combined) (implemented in [ASA] 4.0 software, ANT) as each component is known to have a maximum distribution, notably at central. Therefore, this site was selected and averaged for the amplitude measurements.

For both conditions and each group, we measured the latency of the components of interest from the stimulus onset to the time of the peak maximum, and its amplitude was measured at this peak maximum.

### Statistical Analysis

Demographic and clinical characteristics, as well as differences in cognitive performances (Cogstate tasks), between the patients and controls were assessed using independent sample *t*-tests with unequal variances.

To normalize the variance of the EEG data (i.e., magnitude of ERPs), a square power transformation was used. The normality of ERP data distribution was verified with the Shapiro-Francia test. After transformation, all of these variables were normally distributed. Infrequent tones, group (controls and patients), task condition (control and counting backward), session recording (D0 and D15), age, medication (benzodiazepine equivalent units), and a group × task interaction were included as independent variables in a repeated measure linear regression model to analyze their respective influence on each of the dependent variables (EEG measures). Statistical analyses were restricted to latency and amplitude of the N1, N2, and P3b components for the individual average waveforms. We also adjusted all models for gender.

All statistical analyses were pre-planned in the protocol. We performed statistical analyses using the Stata software package, version 16.1. The statistical threshold for α was set at *p* < 0.05.

## Results

### Demographic and Clinical Results

Demographic characteristics at inclusion did not differ between ADP and controls (Table 1). During neuropsychological testing at D15, some deficits in verbal learning and memory were present in ADP, as compared to controls (Table 3).

### Electrophysiological Results

#### Event-Related Potentials (ERPs)

Analysis of averaged ERPs revealed the presence of the three components previously described in the literature in both detection (workload-free) and counting backward (with workload) tasks.

**Figure 1:**
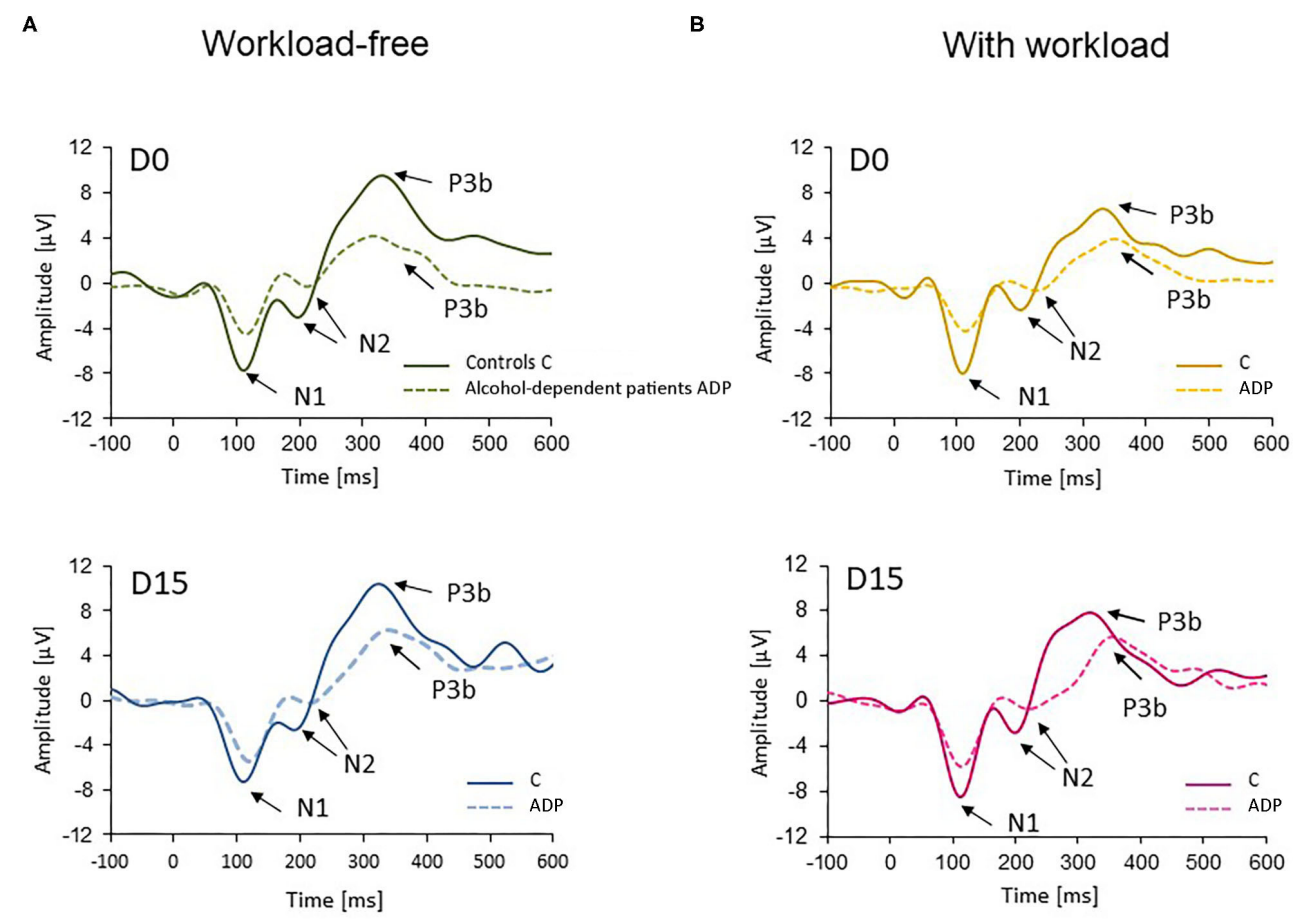
Grand average waveforms at central (C3, Cz, and C4 combined) electrode sites for controls (C, solid lines) and alcohol-dependent patients (ADP, dotted lines) at D0 (upper panels, green and yellow) and D15 (lower panels, blue and pink) during **(A)** the workload-free task, and **(B)** the workload task for infrequent target auditory stimuli.

Figure 1 describes these waveforms at central electrode locations (C3, Cz, and C4 electrodes combined) for all subjects and conditions: the first component N1 consists of an early negative wave with a peak latency around 100 ms after stimulus onset; the second component N2 consists of an early negative wave with a peak latency around 230–280 ms; the third component P3b is a positive component with a peak latency around 320–440 ms.

**Figure 2:**
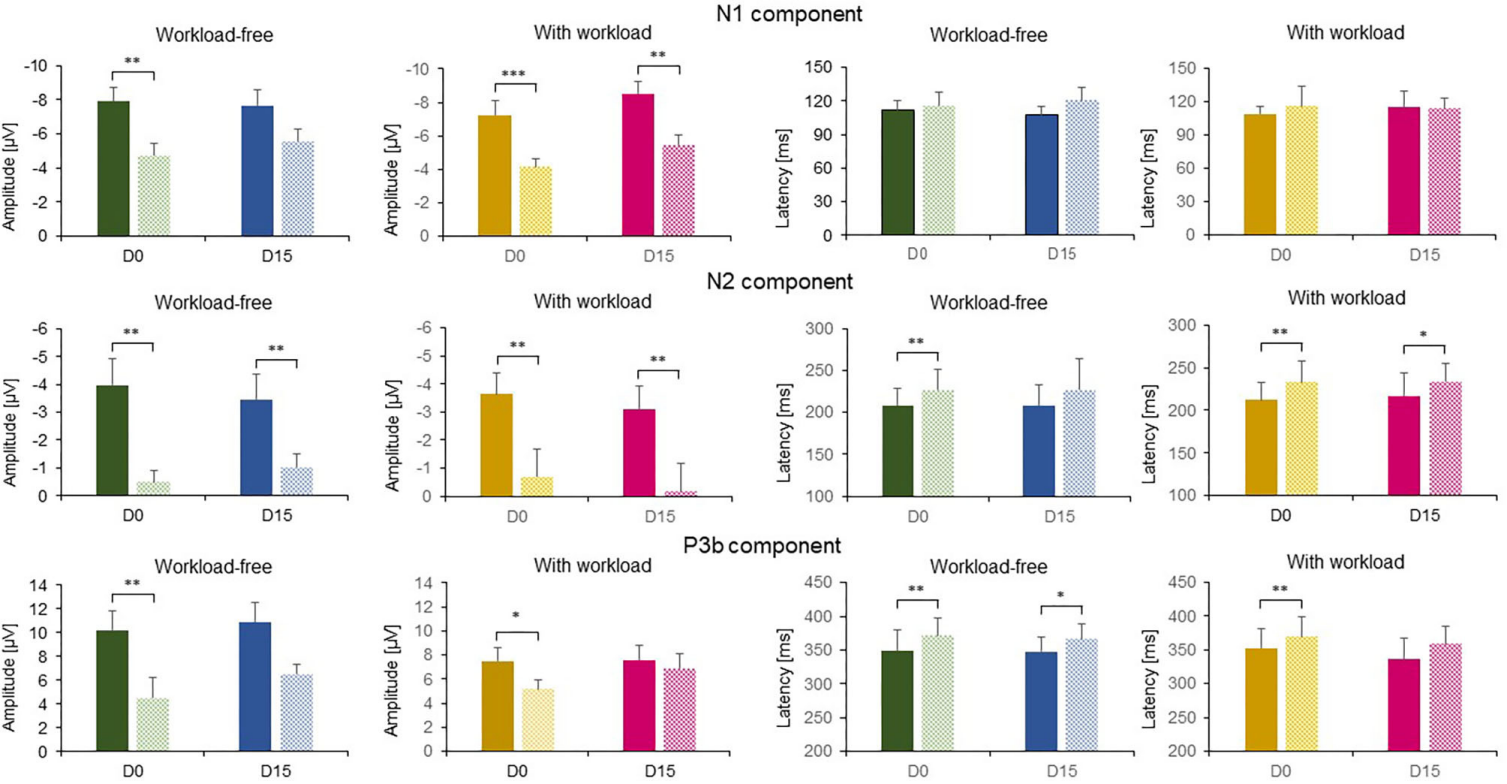
Bar histograms represent averaged amplitude (left) and latency (right) of the ERP waveforms ± SD of controls (C, solid bars) and alcohol-dependent patients (ADP, dotted bars) at D0 (green, yellow) and D15 (blue, pink) for the workload-free and workload tasks for infrequent target auditory stimuli. Asterisks represent statistically significant differences in the linear regression model (*p < 0.05, **p < 0.03, ***p < 0.01).

Table 4 describes all comparison statistics for main related effects and interactions. Figure 2 shows the mean amplitude and latency of each ERP component of interest for all subjects and conditions, as well as *post-hoc* statistics.

**Table 4:** Statistical results of main related effects for exogenous and endogenous ERP latencies [ms] and amplitudes [μV] when controls (*n* = 18) were compared to alcohol-dependent patients (*n* = 18).

**Conditions**																		
	**N1 component**						**N2 component**						**P3b component**					
	**Coef**.	**Std. Err**.	***z***	***P***	**[95% Conf. Interval]**		**Coef**.	**Std. Err**.	***z***	***P***	**[95% Conf. Interval]**		**Coef**.	**Std. Err**.	***z***	***P***	**[95% Conf. Interval]**	
**Amplitude [μV]**
Group effect	3.163	1.019	**3.11**	**0.002**	1.167	5.160	4.475	1.272	**3.52**	**0.001**	1.982	6.962	−5.660	1.697	**−3.34**	**0.001**	−8.986	−2.335
Session recording	0.258	0.695	0.37	0.711	−1.104	1.619	−2.043	1.345	−1.59	0.129	−4.679	0.593	0.698	1.284	0.54	0.586	−1.818	3.214
Task effect	0.666	0.695	0.96	0.338	−0. 696	2.028	−1.524	1.345	−1.13	0.257	−4.160	1.112	−2.700	1.284	**−2.10**	**0.035**	−5.216	−0.184
Session × Task Interaction	−1.514	0.983	−1.54	0.123	−3.430	0.412	0.002	1.345	0.01	0.999	−2.634	2.639	−0.575	1.815	−0.32	0.751	−4.133	2.983
Group × Session × Task interaction	1.057	1.390	0.76	0.447	−1.667	3.781	2.217	1.902	1.17	0.244	−1.511	5.945	2.460	2.567	0.96	0.338	−2.572	7.491
**Latency [ms]**
Group effect	3.630	3.797	0.96	0.339	−3.813	11.072	18.15	8.225	**2.28**	**0.023**	2.623	34.871	22.080	8.646	**2.55**	**0.011**	5.134	39.025
Session recording	−4.667	3.551	−1.31	0.189	−11.627	2.293	0.428	6.600	0.06	0.948	−12.501	13.357	−1.583	6.852	−0.23	0.817	−15.013	11.846
Task effect	−3.389	3.551	−0.95	0.340	−10.349	3.571	4.083	6.597	0.62	0.536	−8.846	17.012	3.033	6.852	0.44	0.658	−10.400	16.463
Session × Task Interaction	10.444	5.021	**2.08**	**0.038**	0.602	20.287	4.772	9.329	0.51	0.609	−13.512	23.057	10.161	9.690	1.05	0.294	−8.831	29.153
Group × Session × Task Interaction	−17.242	7.102	**−2.43**	**0.015**	−31.161	−3.322	−3.689	13.193	−0.28	0.780	−29.547	22.169	15.708	13.703	−1.15	0.252	−42.566	11.151

*The statistical threshold for α was set at p < 0.05. Bold values indicate statistical significance*.

The auditory sensorial N1 component was observed at D0 and D15 for both conditions and both groups. Its amplitude was significantly reduced in ADP compared to controls (*z* = 3.11, *p* = 0.002). No other main effects or interactions between factors were observed. *Post-hoc* analysis showed that the N1 amplitude was renormalized by BZ in the workload-free task.

The N1 latency was free from main related effects. However, there were significant interactions between session recording and tasks (*z* = 2.08, *p* = 0.038) and between groups, session recordings and tasks (*z* = −2.43, *p* = 0.015).

The amplitude of the N2 component was significantly modulated according to the group (*z* = 3.52, *p* = 0.001), revealing a lower amplitude in ADP. This effect was independent of session recording or task condition. No two- or three-way interactions between factors were observed.

The N2 latency was significantly longer in ADP as compared to controls (*z* = 2.28, *p* = 0.023). No other main effects or interactions between factors were observed. *Post-hoc* analysis showed that the N2 latency was renormalized by BZ in the workload-free task.

For the P3b component, the amplitude was significantly lower in ADP as compared to controls (*z* = −3.34, *p* = 0.001). There was also a significant task effect (*z* = −2.10, *p* = 0.035), revealing a lower P3b amplitude in high-workload condition in controls but not in ADP. No session recording effect or two- or three-way interactions between factors were observed. *Post-hoc* analysis showed that the P3b amplitude was renormalized by BZ in both tasks.

The latency of the P3b was significantly increased in ADP as compared to controls (*z* = 2.55, *p* = 0.011). No other main factors or interaction-related effects were observed. *Post-hoc* analysis showed that the P3b latency was renormalized by BZ in the workload task.

Finally, when the regression models were adjusted for gender, this variable was neither statistically significant, nor did it change the results.

## Discussion

The present study contributes to the current debate about the efficacy of rehabilitation treatments on the stages of information processing by the brain, in the context of alcohol withdrawal as well as relapse prevention. Using adapted assessment paradigms, we examined in alcohol-dependent patients the effects of the benzodiazepine-based standard detoxification program on electrophysiological brain responses at incoming (D0) and completion (D15) of the treatment. Using ERPs analysis, we demonstrate that the time-course and the intensity of brain processing events involved in allocation of attention are still impaired at the conclusion of the treatment. This reveals that, even if most clinical scores returned to normal values, subtle neural bases alterations persisted after a 2-week treatment.

We clearly distinguished a N1 component in both groups at a central location around 100 ms after stimulus onset (Figure 1). The N1 mean amplitude was significantly decreased in ADP as compared to controls at inclusion (D0, Figure 2). Although the amplitude of this ERP component was renormalized at the conclusion of the withdrawal treatment for the workload-free task in patients (D15, Figure 2), it remained significantly lower for the workload task. This suggests the persistence of deficits to activate neural bases related to sensory treatment processes during engagement of complex cognitive processes in ADP.

Interestingly, in the workload-free task the renormalization of the N1 amplitude by BZ, which are GABAergic drugs, would thus reflect a restored functionality of the neurons responsible for this component. In line with this idea, it has been described that the generation of the N1 component depends on GABAA receptors activity (34, 35). Acute alcohol consumption induces changes in the subunit composition of GABAA receptors (36), while chronic alcohol intake leads to a desensitization of these same receptors (37). Our data provide evidence that a 2-week GABAergic treatment with BZ in our chronic ADP allowed the activation of networks involved in the normal sensory processing at the pre-attentional stages.

However, the persistence of differences between both groups at the end of the treatment in the workload task strongly suggests that ADP were not able to activate additional neuronal populations involved in a complex cognitive demanding task, such as our workload task. Physiologically, the increase in mental workload during a bizarre paradigm for expected tones engages auditory and frontal cortical generators at the origin of a central negative component that overlaps the exogenous component N1 and shifts its peak of latency to the right (38, 39). Cognitively, this negative component reflects the activity of specific neuronal populations comparing each new stimulus to an attentional trace stored in sensory memory (40). In this context, our findings support the view that neural bases of this component could not be activated in a specific manner in the ADP.

The following component, the N2, was present independently of the task difficulty in controls and ADP patients at incoming and completion of the treatment (Figure 1). Numerous EEG studies have shown an increase of N2 amplitude for the infrequent target tones compared to non-target stimuli in control participants performing auditory oddball tasks (41), revealing that this component surrounds attentional processes associated with the detection of a novel stimulus in the oddball task.

In our ADP, the amplitude of N2 was decreased at their inclusion, and remained so at the conclusion of the treatment (Figure 2). This was previously reported in the literature in this population of patients (42, 43). The most plausible explanation is that ADP may not recruit neural generators in an adapted manner. Several lines of evidence support this hypothesis. First, the latency of the N2 component was longer as well as slightly flatter in both tasks in ADP (Figure 1), suggesting a deficit in the synchronicity of the neurons involved. Second, the amplitude was decreased, suggesting a weaker activation of the neuronal population.

The N2 component, which is frequently interpreted as an index of inhibition and/or conflict control, allows us to assume the location of the deficit in neuronal activation, as it originates from prefrontal regions, specifically from the anterior cingular cortex (ACC). A previous study demonstrated an inadequate commitment from the ACC during an oddball task as the origin of the N2 amplitude in alcoholic patients (44), which is consistent with our results. Analyzing the neuropsychological results revealed a persistence of visual memory and attention deficits at the conclusion of the treatment in ADP. These results are consistent with our ERP results since N2 parameters were not re-normalized. They provide further evidence of the low efficacy of BZ to restore the activation of neural networks involved in the allocation of attention.

Together, our findings on the N2 thus argue in favor of an inefficiency of the GABAergic drug treatment to restore the basic activity of neural generators within the ACC in chronic ADP. Previous researches indicated that the N2 amplitude was also largely dependent on a nigro-striatal dopamine pathway (45, 46). Therefore, treatments involving other neurotransmitters may be considered to try and restore the functional activity of N2 generators in alcohol withdrawal.

Lastly, the late exogenous P3b ERP was observed in both groups and sessions, with two main differences between controls and ADP.

The amplitude of the P3b component of ADP at D0 was lower than in controls whatever the task (Figure 2). This is in line with EEG studies that emphasized a reduction of the P3b amplitude in alcohol abuse individuals within different activation tasks including the oddball paradigm (17). The P3b amplitude returned to normal in ADP at D15, showing that BZ-treated ADP can activate neural networks involved in processes related to target stimuli in an adapted manner.

As for the latency of the P3b component, it was delayed for the ADP in both tasks at inclusion (Figure 1). The latency of this component is known to increase in alcoholics performing an auditory detection task. However, this difference between groups disappeared at the conclusion of the treatment, confirming an adequate timing of P3b–related neural generators activation.

Although alcohol-dependent patients no longer consume shortly after detoxification, about 60% of these patients will later relapse (47). Importantly, the presence of changes in the amplitude of ERPs has been documented in relapsing patients, as compared to controls and non-relapsers (18, 23, 24). A persistent reduction of the P3b amplitude has been shown to be a sensitive predictor of a greater risk of relapse (23, 48, 49). A study revealed that alcohol-dependent patients who remained abstinent after a 3 months follow-up period exhibited a decrease in P3 amplitude as compared to relapsers, in response to alcohol-related images (50). This indicated a lower motivation for alcohol-related stimuli, since the P3 amplitude tags the motivational value for the stimulus. Thus, the P3 was deemed a valuable index of proactive inhibition, that may be related to protection vs. alcohol-related stimuli in abstainers (23, 50). Together, our results demonstrate the efficiency of the BZ treatment to normalize both P3b parameters, suggesting that the 2-week standard withdrawal treatment was sufficient to reestablish the neural networks activity linked to P3b. However, a longer follow-up of our ADP with sequential ERP measurements is mandatory to further conclude on the use of this component as an index of protection to relapse.

Taken together, the present results show that despite a substantial improvement of neural deficits by the detoxification program, the long-standing alcohol-associated neural impairments cannot be totally abolished by an acute cure with BZ. Precisely, alterations in N2 parameters persisted while a renormalization of P3b at D15 for the workload task was observed, indicating only partial neural recovery. The absence of normalization of N2 suggests that this component may play a similar part as the largely reported P3b in alcohol withdrawal. We therefore suggest that the N2 component might also be used to predict treatment outcome. It would be of particular interest to determine if a combination of regular determinations of N2 and P3b in the context of the clinical follow-up would be a useful tool to predict treatment outcome and risk of relapse than using the P3b alone.

A few limitations should be considered when interpreting the present data. First, given that the paradigm was performed in one 30 min-session of recording, we cannot definitively rule out a decreasing attention with time-related fatigue in the ADP. However, we consider that this effect would be negligible since the differences between groups were present also in the workload-free condition, which requires less attention. Second, psychiatric and addictive comorbidities in our ADP might be responsible for some of the results at D0. This unfortunately cannot be excluded, yet the improvement seen at D15 suggests that neuronal plasticity was possible even in a context of poly-addictive behavior. Lastly, the effects observed at D15 could also be partly due to abstinence from alcohol use and to the acute administration of BZ. However, the physiological deficits seen at completion were related to the N2, which is a dopamine-dependent component. Moreover, the short bioavailability of Oxazepam® strongly suggests that the effects of BZ were most probably dissipated during the D15 session recording. Therefore, it is highly unlikely that our results were influenced by BZ treatment.

## Conclusion and Perspectives

In conclusion, using an adapted procedure we investigated the impact of the BZ-based standard pharmacological detoxification treatment on parameters of major brain evoked responses. ERPs that reflect the most important stages of information processing, which is crucial for appropriate decision-making, may be particularly valuable to predict the efficiency of treatment and the risk of relapse. Specifically, the absence of a complete normalization of the P3b and N2-based neurophysiology could be the biological signature behind the limited effectiveness of standard treatments in these patients. Clinically, we thus propose that ERP components should always be investigated during the whole treatment period and at its conclusion, as well as over several months, in order to ensure of the renormalization of the major steps of information processing. ADP with impaired parameters related to both P3b and N2 components should be more closely monitored. Further studies combining multiple assessment of ERPs and neuropsychological investigation are needed in larger series with a longer follow-up. This would allow to elucidate whether this neurophysiological deficit could be used as a direct index of relapse in detoxified alcoholic hospitalized patients.

## Data Availability Statement

The raw data supporting the conclusions of this article will be made available by the authors, without undue reservation.

## Ethics Statement

The studies involving human participants were reviewed and approved by Comité d'éthique Université de Fribourg. The patients/participants provided their written informed consent to participate in this study.

## Author Contributions

PM contributed to the conception and design, the recordings, analysis, interpretation of data, and to drafting and revising the article. GF, DD, and FH contributed to the development of the analyses of the data and to revising the paper. IG and AK contributed to the recruiting, characterization, conception and design of the study, and to revising of article. AP and MM contributed to drafting and revising the article. All authors approved the final version of the manuscript.

## Conflict of Interest

The authors declare that the research was conducted in the absence of any commercial or financial relationships that could be construed as a potential conflict of interest.

## Publisher's Note

All claims expressed in this article are solely those of the authors and do not necessarily represent those of their affiliated organizations, or those of the publisher, the editors and the reviewers. Any product that may be evaluated in this article, or claim that may be made by its manufacturer, is not guaranteed or endorsed by the publisher.
